# MBGapp: A Shiny application for teaching model-based geostatistics to population health scientists

**DOI:** 10.1371/journal.pone.0262145

**Published:** 2021-12-31

**Authors:** Olatunji Johnson, Claudio Fronterre, Peter J. Diggle, Benjamin Amoah, Emanuele Giorgi

**Affiliations:** 1 CHICAS, Lancaster Medical School, Lancaster University, Lancaster, United Kingdom; 2 Department of Mathematics, University of Manchester, Manchester, United Kingdom; Florida Atlantic University, UNITED STATES

## Abstract

User-friendly interfaces have been increasingly used to facilitate the learning of advanced statistical methodology, especially for students with only minimal statistical training. In this paper, we illustrate the use of MBGapp for teaching geostatistical analysis to population health scientists. Using a case-study on *Loa loa* infections, we show how MBGapp can be used to teach the different stages of a geostatistical analysis in a more interactive fashion. For wider accessibility and usability, MBGapp is available as an R package and as a Shiny web-application that can be freely accessed on any web browser. In addition to MBGapp, we also present an auxiliary Shiny app, called VariagramApp, that can be used to aid the teaching of Gaussian processes in one and two dimensions using simulations.

## Introduction

Geostatistical methods were originally developed for applications in the mining industry [1], but are nowadays used in different fields of science, including climatology, geology, agriculture and epidemiology. Diggle *et al*. [2] used the term *Model-based geostatistics* (MBG) to mean the application of stochastic modelling and likelihood-based inference to geostatistical problems, i.e. problems concerning the spatial variation of a phenomenon of scientific interest. Recently, there has been an increasing need for scientists from varied backgrounds to learn and apply geostatistical methods to address substantive scientific problems. In this paper, we shall focus our attention on the use of geostatistical methods for public health problems where the goal is to use spatially discrete cross-sectional survey data to investigate spatial variation in disease risk.

Training of population health scientists in geostatistics poses pedagogical challenges that vary according to the trainee’s prior knowledge of the foundations of statistical and mathematical science. In the authors’ experience, the central challenge is to teach *why* MBG methods are useful tools for population health research and *how* to interpret the results of an MBG analysis, while keeping the mathematical formalism to a minimum. Current advances in technology have revolutionised the way statistics is applied and taught in different settings. The use of user-friendly applications in the lecture room can create an effective learning environment [3] that helps students to understand complex statistical concepts through interactive animations. Another advantage of this approach is that it can avoid the need to teach advanced programming skills, which can be more of a distraction when students want to apply a newly learnt statistical method, especially if they have little or no coding experience. It has been shown that embedding statistical modelling approaches in a user friendly application can help to motivate students to learn and appreciate the use of statistical methods that are new to them [4–6].

Our objective in this paper is to demonstrate the use of a newly developed Shiny application for teaching model-based geostatistics in a course aimed at population health scientists with little or no programming skills and only a basic understanding of statistics. Shiny [7] is a web application framework that has been developed by Rstudio to facilitate the creation of interactive web-based applications in R [8], using dynamic and visually appealing graphics and tables. Making use of these interactive features, MBGapp can be used to introduce key concepts of geostatistical analysis, without requiring any programming skills.

The structure of the paper is as follows. In the Methods, we describe the data format that can be handled in the MBGapp, provide a brief overview of the underpinning statistical methodology and describe the interface of the Shiny application. In the Results section, we provide a step-by-step explanation of how to use the MBGapp for geostatistical analysis using *Loa loa* (eyeworm) prevalence data from Cameroon. The R functions that are used within MBGapp for spatial exploration, parameter estimation and spatial prediction are part of the PrevMap [9] R package. Trainees can therefore “graduate” from MBGapp to direct use of PrevMap as and when they gain experience in using R.

## Methods

In this section we illustrate the type of data that can be analysed with the app, show how to install and use the app, and describe its functionalities.

### Data scenario and modelling formulation

In geostatistical analysis, the data can be distinguished as follows.

- **Location data**: The set of locations at which outcome data are obtained is used to compute the spatial correlation between outcomes at different locations and to extract values of covariates that are available as *raster* data.
- **Outcome data**: Three types of outcome data that are handled by the Shiny app: continuous data; bounded count data, which we refer to as Binomial data; and unbounded counts, or Poisson data. Examples of continuous data include elevation and temperature. An example of a Binomial outcome is prevalence, consisting of the number of people tested and the number who test positive for the disease of interest. Poisson data typically arise from counting the number of newly reported cases of a disease within a given time period.
- **Covariate(s) data**: These are variables that are deemed to be associated with the outcome of interest. Their use in geostatistics is to assist the prediction of the outcome at unsampled locations.

The main ingredients of a geostatistical model are: *Y*_*i*_, the outcome of interest, for *i* = 1, …, *n*, with *n* representing the total number of observations; the locations *x*_*i*_, where the *Y*_*i*_ are taken; covariates, *d*(*x*_*i*_), observed at locations *x*_*i*_, which are associated with a vector of regression coefficients *β*; *S*(*x*_*i*_), a spatial random effect accounting for the unexplained spatial variation in the outcomes; and, finally, unstructured random effects, *Z*_*i*_, that account for unexplained variation within a particular location. An example of the last of these would be when a nominally binomial outcome remains over-dispersed after accounting for covariate effects and unexplained spatial variation.

We adopt a generalized linear modelling framework, in which the *Y*_*i*_, conditionally on *S*(*x*_*i*_) and *Z*_*i*_, belongs to the exponential family, which includes the Gaussian, Binomial and Poisson distributions as special cases. Hence, we specify the conditional mean and variance of *Y*_*i*_, given *S*(*x*_*i*_) and *Z*_*i*_, as *E*[*Y*_*i*_|*S*(*x*_*i*_), *Z*_*i*_] = *m*_*i*_ *μ*_*i*_ and Var[*Y*_*i*_|*S*(*x*_*i*_), *Z*_*i*_] = *m*_*i*_*V*(*μ*_*i*_), where *m*_*i*_ is an offset quantity and *V*(⋅) is a so-called variance function. To fully specify a generalized linear geostatistical model, we then define the *linear predictor* as

$$\begin{array}{c} g(\mu_{i})=d{\left(x_{i}\right)}^{⊤}\beta +S\left(x_{i}\right)+Z_{i}, \end{array}$$ (1)

where *g*(⋅) is a link function. Table 1 shows the expectation, variance function and link function for the implemented cases of the conditional distribution of the outcome. The spatial stochastic process *S*(*x*) is usually modelled as a zero-mean stationary and isotropic Gaussian process with variance *σ*^2^ and Matérn correlation function [10], *ρ*(*u*;*ϕ*, *κ*), where *u* = ‖*x* − *x*′‖ is the Euclidean distance between locations *x* and *x*′, *ϕ* is the scale parameter and *κ* is the smoothness parameter. The *Z*_*i*_, also referred to as the *nugget effect*, are independent and Normally distributed with mean 0 and variance *τ*^2^.

**Table 1:** Summary of the generalized linear geostatistical model distributions implemented in MBGapp: The conditional distribution of *Y*_*i*_ given the random effects *S*(*x*_*i*_) and *Z*_*i*_, including its expectation, variance function and link function.

Conditional distribution	Expectation	Variance function	Link function
Gaussian	*μ*_*i*_	*σ* ^2^	*μ*_*i*_
Binomial	*m*_*i*_ *μ*_*i*_	*m*_*i*_ *μ*_*i*_(1 − *μ*_*i*_)	log(*μ*_*i*_/{1 − *μ*_*i*_})
Poisson	*μ*_*i*_	*μ*_*i*_	log{*μ*_*i*_}

**Fig 1:**
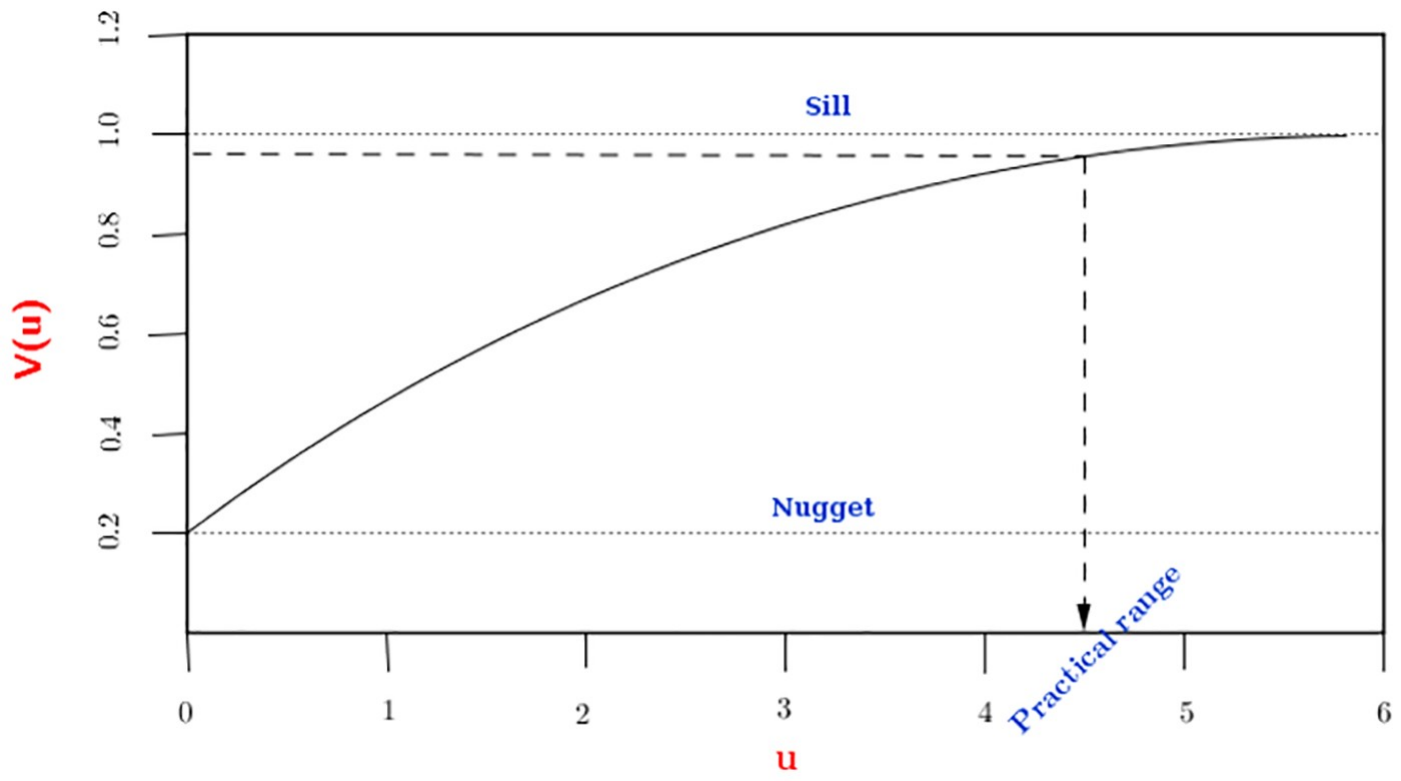
Plot of the theoretical variogram and its main features.

The *theoretical variogram* is a commonly used tool in geostatistics to visualize the spatial structure of the model in (1). This is defined as

$$\begin{array}{c} v\left(u_{ij}\right)=E\left[{\left\{W\left(x_{i}\right)-W\left(x_{j}\right)\right\}}^{2}\right]=\tau^{2}+\sigma^{2}\left(1-\rho \left(u_{ij}\right)\right), \end{array}$$ (2)

where *u*_*ij*_ is the distance between locations *x*_*i*_ and *x*_*j*_ and *W*(*x*_*i*_) = *S*(*x*_*i*_) + *Z*_*i*_ is the sum of the random effects in the linear predictor. The theoretical variogram *v*(*u*) is a non-decreasing function of *u* as shown in Fig 1, which flattens just below the sum *σ*^2^ + *τ*^2^, also known as the *sill*. The *practical range* of the variogram is defined as the distance u* which gives a correlation of 0.05, i.e. *ρ*(u*) = 0.05. These features of the theoretical variogram will be of particular relevance in the next section, where we explain how to investigate residual spatial correlation in the data after taking account of available covariates.

#### Checking for evidence of spatial correlation

The first stage of a geostatistical analysis is to check for the evidence of spatial correlation in order to justify the introduction of the *S*(*x*) term in the model expressed by Eq (1). To do this, we use the empirical variogram, which is the empirical counterpart of the theoretical variogram presented in the previous section. Let ${\hat{r}}_{i}$ denote the residual at location *x*_*i*_ after fitting a standard linear model that excludes *S*(*x*_*i*_) from (1). In the case of Binomial or Poisson data, the linear model is fitted to the empirical logit-transformed counts, log{(*y*_*i*_ + 0.5)/(*m*_*i*_ − *y*_*i*_ + 0.5)}, or log-transformed counts, log{(*y*_*i*_ + 1)/*m*_*i*_}, respectively. Now, for each of *g* = 1, …, *m*, calculate *V*_*g*_ as the sample mean of squared differences $v_{hk}={\left({\hat{r}}_{h}-{\hat{r}}_{k}\right)}^{2}$ from all pairs of locations (*x*_*h*_, *x*_*k*_) whose Euclidean distance ‖*x*_*h*_ − *x*_*k*_‖ falls within the *g*th of a predefined set of distance intervals, usually referred to as *bins*, with centres *u*_*g*_. The *empirical variogram* is a plot of *V*_*g*_ against *u*_*g*_.

In the absence of spatial correlation the expectation of *V*_*g*_ does not vary with *u*_*g*_. A trend in the empirical variogram is therefore interpreted as evidence of spatial correlation. Typically, any such trend is increasing, reflecting a decrease in spatial correlation with increasing distance. However, because of the uncertainty inherent in the empirical variogram, it can often be difficult to assess residual spatial correlation from a simple visual inspection of the empirical variogram. To address this problem, we generate a 95% tolerance envelope around the variogram generated under the assumption of spatial independence, as follows:

1. permute the ${\hat{r}}_{i}$, holding the *x*_*i*_ fixed;
2. compute the empirical variogram, *v*_*hk*_ using the permuted ${\hat{r}}_{i}$;
3. repeat (1) and (2) *L* times (typically *L* = 100 or 1000);
4. use the resulting *L* empirical variograms to generate 95% tolerance intervals in each of the pre-defined bins.

If the empirical variogram lies wholly inside the 95% tolerance envelope generated in step 4, we conclude that there is no spatial correlation. If, instead, the empirical variogram lies partly outside the 95% envelope, we interpret this as evidence of spatial correlation, which warrants the use of a geostatistical model.

#### Inference: Estimation and prediction

The term *estimation* refers to inference concerning the parameters *θ* = (*β*, *σ*^2^, *ϕ*, *τ*^2^) of model (1), whilst *prediction* refers to inference concerning the realisation of the linear predictor.

In MBGapp, estimation uses the maximum likelihood method. The ingredients of likelihood-based inference are: 1) the conditional probability distribution of the data *Y* given *S* as a function of the unknown parameters, *θ* = (*β*, *σ*^2^, *ϕ*, *τ*^2^); and 2) the unconditional distribution of *S*. The likelihood function, written as *L*(*θ*) is then obtained by integrating out *S*,

$$\begin{array}{c} L(\theta )=\int [Y|S;\theta ][S;\theta ]\;dS, \end{array}$$ (3)

The integral is analytically tractable for the Gaussian model; binomial and Poisson models require Monte Carlo methods [11].

The first step in predictive inference is to define a predictive target, *T*(*x*). In MBGapp, the predictive targets that are available are: *T*(*x*) = *d*(*x*)^⊤^ + *S*(*x*) for the Gaussian distribution; T*(*x*) = exp{*d*(*x*)^⊤^ + *S*(*x*)}/(1 + exp{*d*(*x*)^⊤^ + *S*(*x*)}) for the Binomial distribution; and *T*(*x*) = exp{*d*(*x*)^⊤^ + *S*(*x*)} for the Poisson distribution.

To make inferences about *T*(*x*), we use its predictive distribution, defined as the distribution of *T*(*x*) conditional on the data *y*_*i*_, for *i* = 1, …, *n*. In MBGapp, prediction is carried out by fixing the model parameters at their corresponding maximum likelihood estimate. For the details on how the sample from the predictive distribution is carried out, we refer the reader to Section 2.5 of [9].

Summaries of the predictive distribution of *T*(*x*) that can be visualized as a map in MBGapp include the mean, standard error, quantiles and exceedance probabilities. The standard error is a convenient measure of uncertainty when the predictive distribution of each *T*(*x*) is approximately Normal. Otherwise, a pair of quantile maps, for example 5% and 95% quantiles, are to be preferred. An exceedance probability map, i.e. the predictive probability for each location *x* that *T*(*x*) exceeds a fixed value, *c*, is particularly relevant when *c* is a policy-relevant threshold indicating the need for an intervention of some kind; in the public health context see, for example, [12, 13].

### Installation

The web-application can be used online or by installing it as an R package. For the online version, the web address is https://olatunjijohnson.shinyapps.io/mbgapp/.

To use the web application in R, the following steps should be carried out.

1. Download and install R from the Comprehensive R Archive Network (CRAN) on www.r-project.org.
2. For a better user experience, we recommend to download and install RStudio desktop, available at www.rstudio.com.
3. Finally, install MGBapp by running the following line of code in R:
  devtools::install_github(“olatunjijohnson/MBGapp”,ref=“main”)

**Fig 2:**
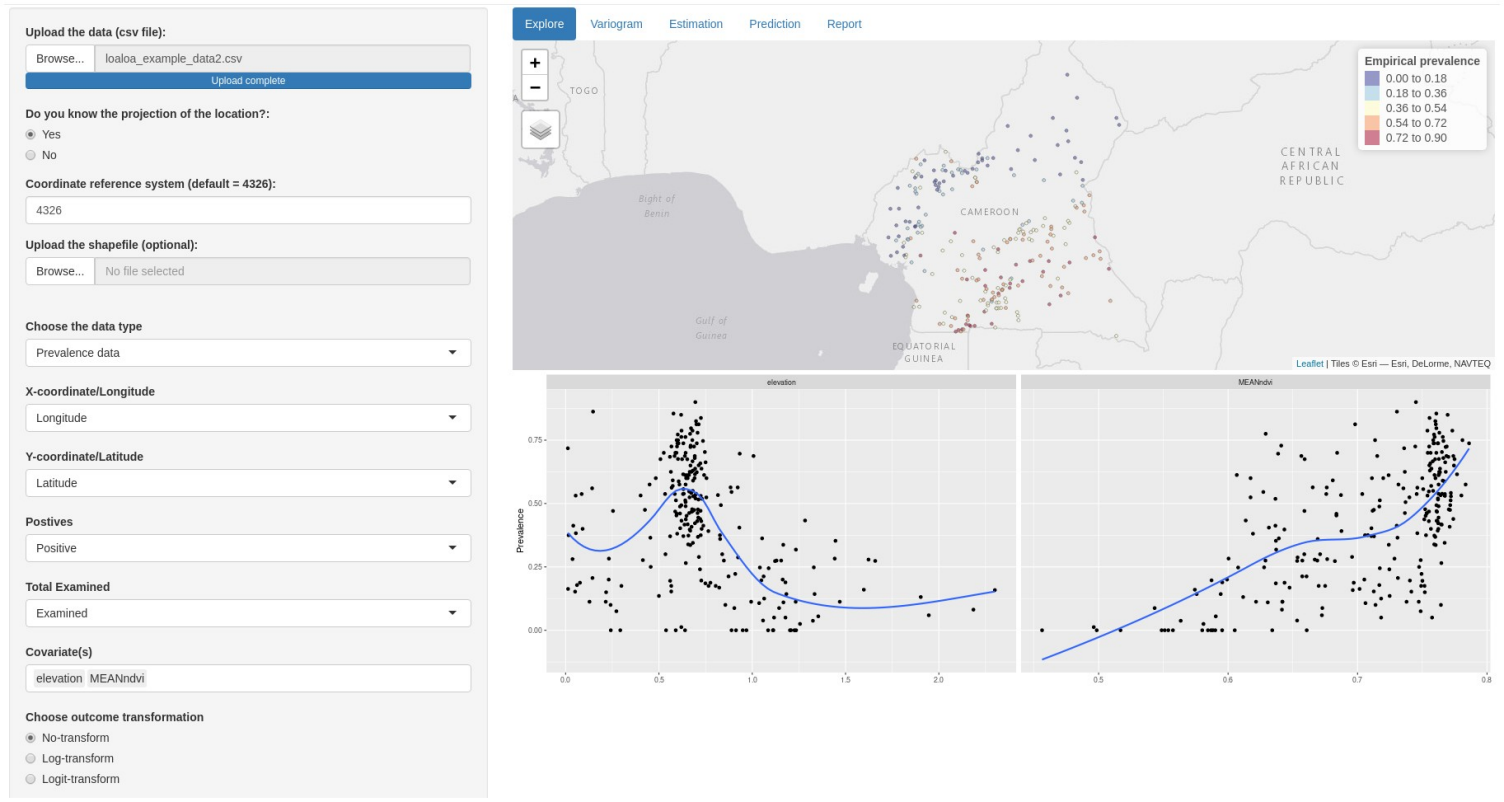
Exploratory analysis. Map showing the empirical prevalence and the locations of the villages surveyed (upper panel) and the scatter plot of the relationship between the prevalence and elevation and NDVI.

Once MBGapp has been installed, this can be loaded in R, by typing library(MBGapp) and run_app(), which will open a window as shown in Fig 2.

### Components of the app

A standard Shiny application consists of the sidebar, where the input of the application is controlled, and the main area, where the outputs are displayed. In MBGapp, the main areas comprise four tabs: “Explore”, “Variogram”, “Estimation” and “Prediction”. These four tabs correspond to the steps of a geostatistical analysis that we describe in more detail in the “Results” section. Here, we first describe the functionalities of the sidebar for the different tabs of the main area.

#### Sidebar: Uploading data and defining its format

The sidebar contains the menu that allows users to upload a dataset, a shapefile and enter values or text that control other inputs. To use MBGapp, the first stage is to upload the data and specify their format using the sidebar containing the following fields.

- **Dataset**: The uploaded dataset must be a .csv file containing columns corresponding to the locations (e.g. longitude and latitude) of the data points and the observed measurement (outcome). The dataset can also contain additional variables that are used as covariates, if these are available. Note that in the case of prevalence, information on the outcome is contained in two columns, one corresponding to the number of tested individuals (i.e. the Binomial denominators) and the total number of positively tested individuals at each location.
- **Coordinate reference system**: This field allows the user to specify the coordinate references system (CRS) of the locations in the dataset. MBGapp also allows to carry out a geostatistical analysis, when information on the CRS is missing by answering “No” to the question “Do you know the projection of the location?”. However, whenever this information is available it should always be correctly specified to avoid any ambiguity in the interpretation of the geostatistical model. The CRS provides a standardized way of describing locations, and in the case of longitude/latitude coordinates 4326 is the code that should be provided in the field “Coordinate reference system”.
- **Shapefile (optional)**: Uploading of a shapefile is optional in MBGapp, and it is required only for 1) showing the boundary of the study of interest on map and 2) generating the predictive grid for the spatially continuous predictions of the outcome. To upload the shapefile, the user can click on “Browse” button and select all the shapefile files extensions (.dbf, .prj, .shp, .xml and .shx) from the folder stored on their personal device.
- **Type of dataset**: This allows the user to specify the type of outcome to be analysed: continuous data, prevalence data and count data. Selecting the appropriate option here allows MBGapp to identify the corresponding geostatistical model used in the analysis.

#### Exploration tab

The Explore tab is where the user can visualise the uploaded data on a map and explore the relationship between the outcome and the available covariates.

1. **Mapping the outcome**: to visualize the data as a point-map after specifying the data-type, two fields needs to be specified: “X-coordinate/Longitude” and “Y-coordinate/Latitude”. The columns corresponding to the outcome data should then be specified: if the outcome is continuous, only one field is required; for Binomial and Poisson data, the outcome is specified in two fields. For example, in the case of prevalence data, one field identifies “Positives” and another “Total examined”. After these are specified, all the other menus in MBGapp are automatically updated using the same column names specified at this stage. Once the data are visualized on a map, it is possible to change the base map using the layer control icon, where the following options can be selected: “Esri.WorldGrayCanvas”, “OpenStreetMap” and “Esri.WorldTopoMap”. The default is “Esri.WorldGrayCanvas”.
2. **Understanding the relationship**: Scatter plots are simple graphical tools that are used to understand the empirical relationship between two variables. On the sidebar, the user can specify names of variables under “Covariate(s)” that are used as predictor variables in the geostatistical model. For each of the covariates specified, a scatter plot is then displayed in the main area. Furthermore, in each scatter plot, a smoothing curve obtained using local polynomial regression fitting (loess) [14] is added in order to better understand the functional form expressing the association between the two variables. A range of different transformations of the outcome and the covariate(s) can also be selected in the side bar which can help, for example, to assess the scale on which associations can be modelled as approximately linear. If a non-linear relationship is observed, the “personalised transformation” menu can be used to supply the appropriate function of the covariate such as spline (bs), polynomial (poly), etc.

#### Variogram tab

The Variogram tab is used to examine the residual spatial variation using standard linear regression methods. This tab allows to perform three main tasks: 1) to visualise the residual for the evidence of spatial dependence through the plot of the empirical variogram; 2) to interpolate empirical variograms with theoretical variograms; 3) to test for the evidence of spatial dependence using a permutation test.

To generate an empirical variogram, the following entries need to be specified in MBGapp: “Number of bins” and “Distance”, with the latter defining the maximum distance used for the calculation of the variogram. The field “Correlation functions” provides the available covariance functions including Matern, exponential, Gaussian, spherical, circular, cubic, wave, power-exponential, Cauchy, Gneiting and pure-nugget; and “choose plot” instructs the app what to visualise. Options are variogram only, variogram with envelope, and thereotical variogram overlayed on the sampled variogram.

At the bottom of the variogram tab, the “Learn more” button is a link to another Shiny app for carrying out simulations of Gaussian processes in one and two dimensions, named “VariogramApp”. More details on this separate Shiny app, which we have integrated in MBGapp, can be found in the supplementary material.

#### Estimation tab

The Estimation tab allows the user to carry out parameter estimation of geostatistical models using the maximum likelihood method. To perform estimation, initial guesses of the parameters of the covariance function (Matern) are required. These are the scale parameter, the ratio between the variance of the nugget effect and the Gaussian process, and the the smoothness parameter. The button “Advanced options” allows the users to set the Monte Carlo simulations that are carried out in the case of Binomial and Poisson geostatistical models, namely the number of simulations, burn-in (i.e. number of initial samples that are discarded) and thinning (i.e. frequency used for the storage of separate MCMC samples). For example, with 10,000 simulations, burn-in of 2000 samples and thinning set to 8, would result in a final sample of 1000 samples. “Advanced options” can also be used to include non-linear terms in the model.

After parameter setting is completed, “Show the result summary” can be used to run the model and the “Show table” can be used to display the results in the form of a table.

#### Prediction tab

The Prediction tab is to carry out spatial predictions and visualize maps of the chosen predictive target. In carrying out the predictions, the parameters of the geostatistical model are fixed to the maximum likelihood estimates. The menus of the prediction tab are the following: “Upload the predictive grid”, where the user can upload a csv file of the locations where spatial prediction should be carried out; “Upload the predictors”, where the user can upload a csv file containing the values of the predictors at the locations; “Choose map” allows the user to choose a summary of interest for the spatial predictions, namely mean, standard errors, exceedance probability and quantile. Once all the options from these menus have been selected, the user can finally press the “Map the prediction” button to carry the predictions. If the options “Exceedance probability” or “Quantile” are selected, a slider is then also displayed in order to vary the exceedance probability threshold or quantile.

To use an intercept-only model—i.e. models that do not use any covariates—the user should not upload any covariates at this stage. Also, the user can perform prediction without any predictive grid. In this case, MBGapp creates a prediction grid using either the shapefile boundary, if this has been previously uploaded, or, if that has not been provided, it creates a prediction grid within the convex hull of the sampled locations.

## Results

### Example: Geospatial analysis of *Loa loa* prevalence in Cameroon

The data used for this example are from a cross-section survey conducted in Cameroon on *Loa loa*, also known as “eye-worm” disease. The objective of the analysis is to predict prevalence of *Loa loa*. This data-set can be freely downloaded from the web portal of the “Expanded special project for elimination of neglected tropical disease” (ESPEN), via this link https://drive.google.com/uc?export=download&id=1nGRuw-UUFYbG0Wl4XD4noxaNb1iyyky9. In our example, we use elevation and Normalized Difference Vegetation Index (NDVI) as covariates to assist the prediction of prevalence. The predictors and the 10km by 10km predictive grid can be downloaded from https://drive.google.com/uc?export=download&id=1fe5i08xIghPP_kHUHiL0SNT1usa8L-OD and https://drive.google.com/uc?export=download&id=1-P880pttLqOlyQnW6_K1MPsEQo_Gh4zM, respectively.

#### Step 1: Problem statement

The control of onchocerciasis relies on mass drug administration (MDA) with ivermectin in endemic countries. However, one of the main challenges has been serious adverse effect that has arisen from administering ivermectin to people that are co-infected with *Loa loa*. To make safe ivermectin treatment decisions for onchocerciasis in areas where *Loa loa* is potentially co-endemic, it is important to identify areas of high *Loa loa* prevalence parasites. A threshold of 20% *Loa loa* prevalence is used to exclude areas from the MDA. The objective of the geostatistical analysis will thus be to identify areas that are likely to exceed a 20% prevalence threshold.

#### Step 2: Exploratory analysis

In the exploratory analysis, we visualise the data on a point map and explore the association of *Loa loa* prevalence with elevation. As shown in Fig 2 the empirical relationship between elevation and prevalence is quite noisy and exhibits a non-linear relationship. However, the use of the spline function suggests that *Loa loa* prevalence reaches its maximum at around 0.6km from sea level and decreases thereafter. Also, the empirical relationship between NDVI and prevalence shows a linear relationship.

#### Step 3: Checking for the evidence of spatial dependence

**Fig 3:**
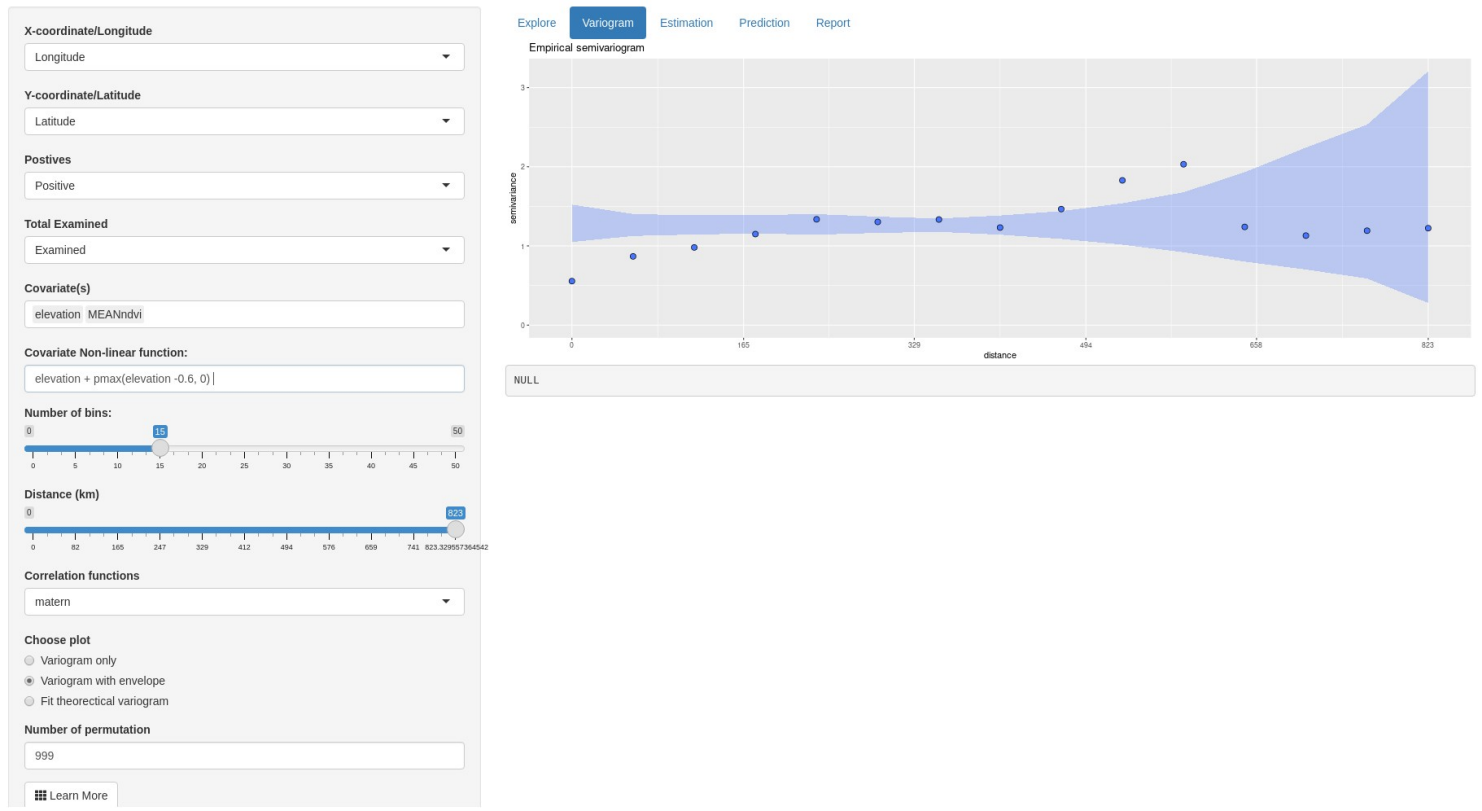
Variogram. Plot showing the empirical variogram and the envelope for confirming the evidence of spatial dependency.

We use the variogram to examine the residual for the presence of residual spatial correlation after fitting a non-spatial model. As shown in Fig 3, the rising trend shows an evidence of spatial correlation. We then use a Monte Carlo strategy as a confirmatory test to establish whether the pattern in the variogram is or not compatible with random fluctuations. Therefore, a simulation of the behaviour of the empirical variogram under the assumption of spatial independence is normally used to construct the envelope. Clearly, there is evidence of residual spatial variation since the empirical variogram lies partly outside the envelope.

#### Step 4: Estimation and prediction

Let the prevalence survey conducted in a village at a geographical location *x* generate data in the form of a pair of values; *n*, the number of individuals surveyed; and *y*, the number of people that tested positive. The sampling distribution of *y* is binomial with number of trials *n* and probability of positive outcome *P*(*x*), the village-wide prevalence at *x*.

**Fig 4:**
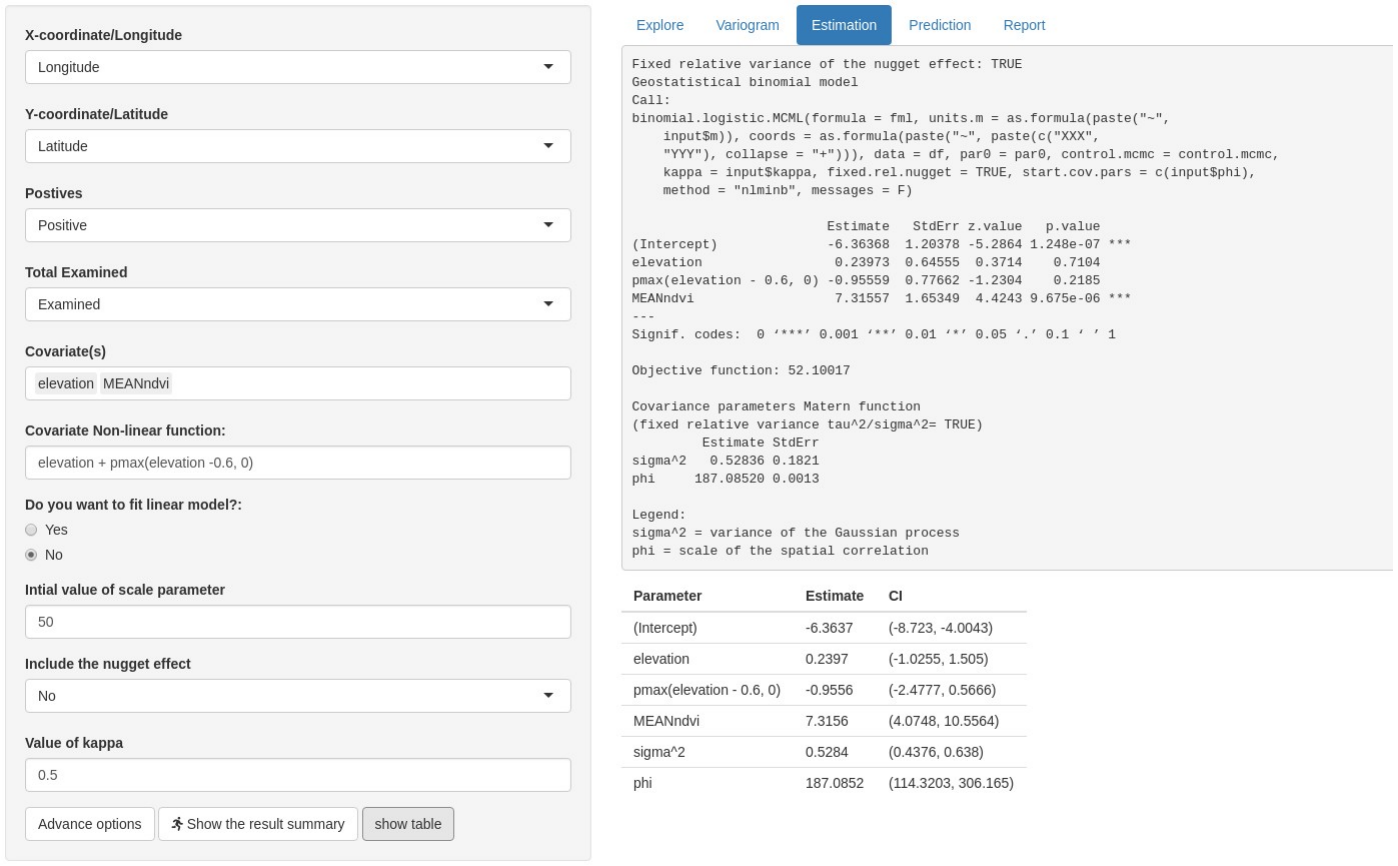
Parameter estimate. The Monte Carlo maximum likelihood estimate as an output of the model (upper panel) and as a table (lower panel) and its corresponding standard error and confidence interval, respectively.

**Fig 5:**
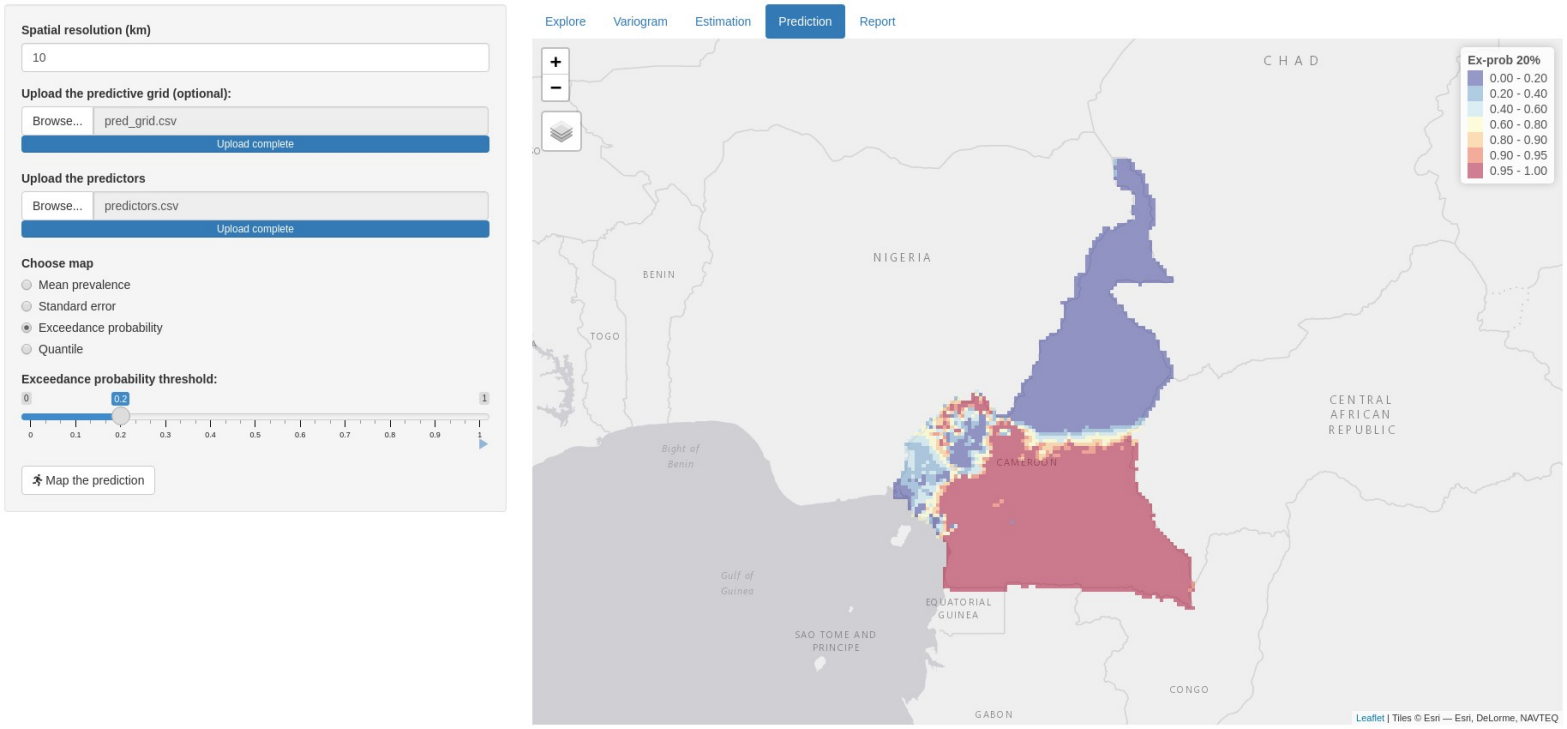
Spatial prediction. Map showing spatially continuous prediction of the probability that prevalence exceeds 20% threshold over a grid of 10km by 10km.

We model the variation in *P*(*x*) using elevation, Elev(*x*) and NDVI(*x*), covariates. The linear predictor of the Binomial geostatistical model takes the form

$$\text{log}[P(x)/\{1-P(x)\}]=\beta_{0}+\beta_{1}\text{Elev}(x)+\beta_{2}\text{max}\{\text{Elev}(x)-0.6,0\}+\beta_{3}\text{NDVI}(x)+S(x),$$ (4)

where max{Elev(*x*) − 0.6, 0} is used to define a linear spline with a change point at 0.6km and thus accounts for the non-linear relationship between prevalence and elevation (Fig 2). We use Monte Carlo maximum likelihood for parameter estimation and the result is shown in Fig 4. We find a positive (*β*_1_ = 0.2397) association between prevalence and elevation less than 0.6km and a negative (*β*_1_ + *β*_2_ = −0.7159) ssociation between prevalence and elevation greater than 0.6km, albeit this is not statistically significant at the conventional 5% significant level. We find a positive association between prevalence and NDVI (*β*_3_ = 7.3156). We also note that the estimate of the signal variance, *σ*^2^ = 0.5284. Finally, the scale of the spatial correlation is about 187km implying a relatively large practical range of about 560km. The parameter estimates are used to carry out prediction at 10km spatial resolution and the predicted exceedance probability surface with 20% threshold is shown in Fig 5. Here, we can distinguish two large areas, one in the south and one in the north of Cameroon that are associated with a very high and very low likelihood of exceeding and not exceeding a 20% prevalence threshold, respectively.

## Discussion

In this paper, we have introduced MBGapp, a user-friendly web application to facilitate the teaching and learning of geostatistical methods for a target audience consisting of population health scientist with limited or no programming skills.

At the time of writing, we have used MBGapp in an online workshop on model-based geostatistics for public health attended by about 25 participants from different educational background, including public health scientists and bio-statisticians. It was found that participants with a good prior knowledge of elementary linear regression and basic probability theory were able to effectively use and understand the different components of the application, with minimal guidance from the lecturers. In the received feedback, about 72% found the usefulness of MBGapp for learning of geostatistical concepts to be “Good” or “Very good”.

We emphasize that MBGapp should be used only for teaching purposes and it cannot handle data with more complex structure than those described in this paper. As part of future work, we aim to extend some of the functionalities of MBGapp for teaching more advanced topics of model-based geostatistics, including handling of zero-inflated data and spatio-temporal analysis.

## Supporting information

S1 Appendix (PDF)

S1 Data (ZIP)
