# Supplementary material for: MBGapp: A Shiny application for teaching model-based geostatistics to population health scientists
Source: PLoS One. 2021 Dec 31;16(12):e0262145. doi: 10.1371/journal.pone.0262145 (PMC8719748; doi:10.1371/journal.pone.0262145)
Supplement: S1 Appendix — (PDF) [file pone.0262145.s001.pdf]

Supplementary material for "MBGapp: A Shiny application for teaching model-based geostatistics to population health scientists"

Olatunji Johnson[1,2*], Claudio Fronterre[1], Peter J Diggle[1], Benjamin Amoah[1], Emanuele Giorgi[1]

1 CHICAS, Lancaster Medical School, Lancaster University, Lancaster, UK
2 Department of Mathematics, University of Manchester, Manchester, UK

VariogramApp: Understanding variogram and Gaussian process simulation

The variogramApp is a separate web application that can be used to assist the students' learning of the the theory of Gaussian processes and how to interpret variogram plots. In particular, it allows users to visualize the spatial patterns and smoothness of Gaussian processes that arises from a given covariance function and set of parameters, and how these are reflected in the variogram. The app provides simulation tools both in one and two dimensions. To access this variogram app in R, the developmental version on GitHub can be run by entering `shiny::runGitHub(repo="variogramApp", username= "olatunjijohnson", ref="master", subdir = "inst/variogramApp")` or by installing the **variogramApp** using

```
devtools::install_github("olatunjijohnson/variogramApp",
                         ref="master").
```

After installing the app, it can loaded in R using

```
library(variogramApp)
```

and then using the command `run_variog_app()`.

The main area of the app displays two figures. One, a simulated surface of $S(x)$ or $S(x) + Z(x)$, where $S(x)$ is a zero-mean isotropic and stationary Gaussian process with $\text{Cov}(S(x), S(x')) = \sigma^2\rho(u, \theta)$, where σ^2 is the variance, $\rho(u, \theta)$ is the correlation function with parameter θ and u is the Euclidean distance between location x and x'; and $Z(x)$ is independent and identically distributed Gaussian noise with nugget variance τ^2. Two, the semi-variogram plot describing the spatial dependence in the data and giving a sense of the estimate of the autocorrelation structure of the underlying stochastic process.

The following is the description of the menus in the sidebars of the app.

1. **Correlation functions:** A number of correlation functions are provided in the app. Let $\theta = (\phi, \kappa)$ denote the vector of the correlation parameters, where ϕ is the scale parameter, κ is the smooth parameter. The correlation functions are as follows:

   - **Exponential**:
   $$C(u;\theta) = \sigma^2 \exp(-u/\phi)$$
   - **Matern**:
   $$C(u;\theta) = \sigma^2(1/(2^{\kappa-1}\Gamma(\kappa)))(u/\phi)^{\kappa})K_{\kappa}(u/\phi),$$
   where $K_{\kappa}(\cdot)$ is the modified Bessel function of the second kind of order $\kappa > 0$.
   - **Gaussian**:
   $$C(u;\theta) = \sigma^2 \exp(-u/\phi)^2$$
   - **Spherical**:
   $$C(u;\theta) = \sigma^2(1 - 1.5(u/\phi) + 0.5(u/\phi)^3) \quad \text{if } u \leq \phi$$
   - **Circular**:
   $$C(u;\theta) = \sigma^2(1 - \gamma(u)) \quad \text{if } u \leq \phi, \ 0 \text{ otherwise}$$
   $$\gamma(\epsilon) = 2((\epsilon * \sqrt{1-\epsilon^2} + \sin^{-1}\sqrt{\epsilon}))/\pi \text{ and } \epsilon = min(u/\phi, 1)$$
   - **Cubic**:
   $$C(u;\theta) = \sigma^2(1-(7((u/\phi)^2)-8.75((u/\phi)^3)+3.5((u/\phi)^5)-0.75((u/\phi)^7)))$$
   $$\text{if } u \leq \phi, \ 0 \text{ otherwise.}$$
   - **Wave**:
   $$C(u;\theta) = \sigma^2(\phi/u)\sin(u/\phi)$$
   - **Power**:
   $$C(u;\theta) = \sigma^2(A - u^{\phi}),$$
   where A is a constant.
   - **Powered exponential**:
   $$C(u;\theta) = \sigma^2 \exp(-u/\phi)^{\kappa} \quad \text{if } 0 < \kappa \leq 2.$$
   - **Cauchy**:
   $$C(u;\theta) = \sigma^2[1 + (u/\phi)^2]^{-\kappa}.$$
   - **Gneiting**:
   $$C(u;\theta) = \sigma^2(1+8su+25s^2u^2+32s^3u^3)(1-su)^8 \quad \text{if } 0 \leq s, \ u \leq 1, \ 0 \text{ otherwise.}$$
   - **Pure nugget**:
   $$C(u;\theta) = \sigma^2, k$$
   where k is a constant value. This model corresponds to no spatial correlation.

2. **Dimension:** The user has the option of simulating the Gaussian process in one or two dimension.

3. **Domain:** Depending on the dimension selected, we provided the options for the users to choose the domain of interest. For example, in one-dimension, the user can choose one of 500, 1000 and 1500 time points while in two-dimension, if the user select a 150 by 100 domain, this implies a x-axis ranging from 0 to 150 and y-axis ranging from 0 to 100.

4. **Variance parameter, σ^2:** The variance parameter must be greater than zero.

5. **Scale parameter, σ^2:** The scale parameter regulates the rate at which the spatial correlation decays for increasing distance u.

6. **Nugget variance, τ^2:** The variance of the nugget effect can be specified here if the user want to include a nugget in the simulation. Setting the nugget variance to zero implies no nugget effect.

7. **Smoothness parameter, κ:** The shape or smoothness parameter determines the differentiability of the process $S(x)$.

8. **Sampling method:** We provides two methods of sampling; random and regular sampling. Random sampling select locations in a random manner while regular sampling imposes a minimum distance between any two sampled locations, therefore introducing minimum distance parameter δ for setting this distance.

9. **Number of samples:** This relates to the number of samples for the construction of the variogram.

10. **Distance:** This is the distance extent of the variogram. A pragmatic choice is about one-third of the maximum extent of the domain of interest as we would not expect any correlation beyond this distance.

11. **Number of bins:** This is the number of classes to average the empirical variograms.

12. **Number of simulation:** This is used by the user to perform this experiment multiple times. If the number of simulations is more than 1 then there is an option to play the result.

13. **Perform Simulation:** The button performs the operation.

Example

Figure 1 shows the result of the default parameters of the app, with exponential correlation function; two-dimension; domain 150 by 100; $\sigma^2 = 1.8$; $\phi = 10$; $\tau^2 = 0.02$; random sampling; 200 samples; 60 as the maximum distance, 15 number of bins, and 1 simulation.

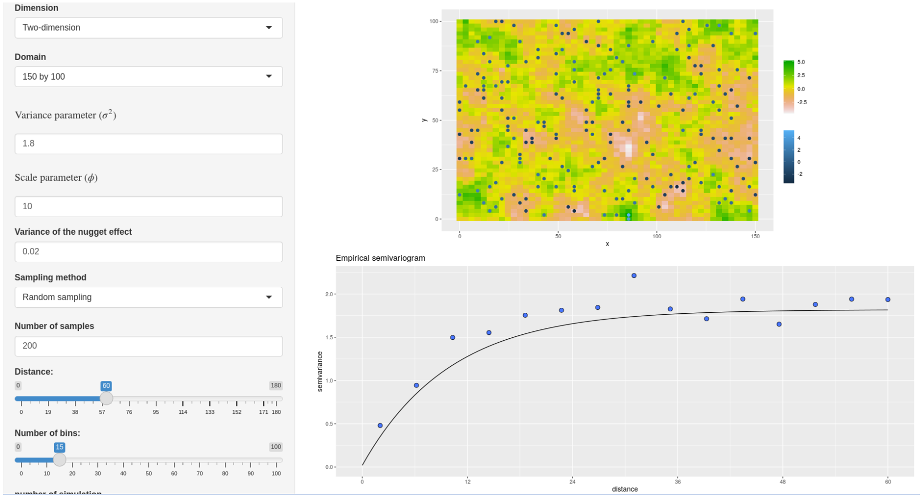

Figure 1: **VariogApp.** The interface of the variogramApp showing the simulated surface and the variogram.
